# *Bacopa monniera* (CDRI-08) Upregulates the Expression of Neuronal and Glial Plasticity Markers in the Brain of Scopolamine Induced Amnesic Mice

**DOI:** 10.1155/2015/837012

**Published:** 2015-08-27

**Authors:** Arpita Konar, Akash Gautam, M. K. Thakur

**Affiliations:** ^1^Biochemistry and Molecular Biology Laboratory, Department of Zoology, Banaras Hindu University, Varanasi 221005, India; ^2^Center for Neural and Cognitive Sciences, University of Hyderabad, Hyderabad 500046, India

## Abstract

Preclinical studies on animal models have discerned the antiamnesic and memory-enhancing potential of *Bacopa monniera* (Brahmi) crude extract and standardized extracts. These studies primarily focus on behavioral consequences. However, lack of information on molecular underpinnings has limited the clinical trials of the potent herb in human subjects. In recent years, researchers highlight plasticity markers as molecular correlates of amnesia and being crucial to design therapeutic targets. In the present report, we have investigated the effect of a special extract of *B. monniera* (CDRI-08) on the expression of key neuronal (BDNF and Arc) and glial (GFAP) plasticity markers in the cerebrum of scopolamine induced amnesic mice. Pre- and postadministration of CDRI-08 ameliorated amnesic effect of scopolamine by decreasing acetyl cholinesterase activity and drastically upregulating the mRNA and protein expression of BDNF, Arc, and GFAP in mouse cerebrum. Interestingly, the plant extract *per se* elevated BDNF and Arc expression as compared to control but GFAP was unaltered. In conclusion, our findings provide the first molecular evidence for antiamnesic potential of CDRI-08 via enhancement of both neuronal and glial plasticity markers. Further investigations on detailed molecular pathways would encourage therapeutic application of the extract in memory disorders.

## 1. Introduction

In the present era, medicinal efficacy of plant extracts mentioned in Ayurvedic literature with large variety of health promoting effects and minimal adversities is on the verge of gaining priority over conventional pharmacological approaches [1]. Among the plants mentioned in Ayurveda,* Bacopa monniera* (Brahmi) stands out in regard to its enormous potency to rejuvenate the central nervous system and improve mental health. The crude extracts of* B. monniera* as well its active principles, that is, bacosides, are being explored in a wide variety of central nervous system disorders, particularly impairment in memory or amnesia [2]. In this context, several studies on animal models [3] including the widely used cholinergic antagonist, scopolamine induced amnesia [4, 5] have revealed the efficacy of* B. monniera* extract in recovering memory impairment and exerting neuroprotection against cholinergic degeneration [6, 7]. The herbal extracts also facilitate short- and long-term memories and memory phases of acquisition, consolidation, and retrieval. However, the underlying molecular mechanisms that sustain the antiamnesic and memory-enhancing potential of* B. monniera* extract are largely unexplored. Consequently this lack of understanding in molecular details has limited the clinical application of* B. monniera* extracts in memory disorders.

Over the past decade, it is considered that the ability of brain to adapt or modify itself in response to intrinsic signals and external environment referred to as plasticity is integral to memory formation. It is a complex dynamic process involving adaptation of intrinsic properties of neurons and glia, reorganization of synaptic connections and neural network [8]. Accumulating evidences have substantiated the role of brain plasticity as a molecular correlate of memory processes [9]. Activation of gene expression associated with neuronal and glial plasticity is considered to be a key mechanism underlying the enduring modification of neural networks required for the formation of memory [10, 11]. Amongst neuronal plasticity markers, brain-derived neurotrophic factor (BDNF), a member of the neurotrophin family, holds the most prominent place. BDNF regulates survival and differentiation of neurons and synaptic plasticity and is considered as a marker of memory decline during pathological conditions and brain aging [12, 13]. Memory acquisition and consolidation are associated with an increase in BDNF expression and activation of tyrosine receptor kinase (TrkB). In several behavioral paradigms including Morris water maze, contextual fear, and passive avoidance, memory formation is associated with a rapid and transient increase in BDNF mRNA expression in the hippocampus. Genetic as well as pharmacological deprivation of BDNF or TrkB impairs long-term potentiation (LTP) and hippocampus-dependent spatial memory [14–16]. BDNF regulates neuronal plasticity by stimulating gene transcription, activating protein synthesis, promoting neurotransmitter release, and modulating the activity and trafficking of postsynaptic receptors. Recently it has been reported that BDNF controls the transcription of neuronal immediate early gene Arc (activity regulated cytoskeletal-associated protein) which is strongly implicated in neuronal plasticity and memory [17, 18]. Several studies demonstrate involvement of Arc in AMPA receptor trafficking, LTP and consolidation of long-term memories [19]. Moreover, Arc knock-out mice showed significant impairment in long-term depression (LTD) and hippocampus-dependent spatial memory [20–22]. Our laboratory also demonstrated the involvement of hippocampal Arc in amnesia [23].

In addition to neuronal plasticity, astroglia and their resident protein glial fibrillary acidic protein (GFAP) are also important for memory function. GFAP is a specific astrocyte marker participating in the formation of cytoskeletal filaments of glial cells and processes of myelinization, cell adhesion, growth of neurites, and synaptogenesis. GFAP is a marker of glial plasticity regulating the astrocyte morphology, neuron-glia interactions, and mechanisms of memory formation [24, 25]. GFAP is drastically downregulated during amnesic conditions [26].

With this background, the present study was designed to investigate the molecular basis of recovery potential of* B. monniera* in scopolamine induced amnesic mouse model focusing on neuronal plasticity markers BDNF and Arc and glial marker, GFAP. The standardized bacoside-A enriched extract of* B. monniera* (CDRI-08) was used in the present study. Initially, antiamnesic potential of CDRI-08 was evaluated by analyzing its effect on scopolamine impaired cholinergic system via acetyl cholinesterase (AChE) activity assay in mouse cerebrum. Thereafter, molecular investigations were performed by examining the effect of CDRI-08 on the mRNA and protein expression of BDNF, Arc, and GFAP in the cerebrum of scopolamine injected amnesic mice.

## 2. Materials and Methods

### 2.1. Chemicals/Reagents

Random hexanucleotides, dNTPs, enhanced chemiluminescence (ECL) reagents, Taq polymerase, RNase inhibitor, and reverse-transcriptase enzymes were obtained from the New England Biolabs (USA); DTNB, acetyl thiocholine iodide, TRI reagent, monoclonal anti-*β*-actin-peroxidase (A3854) and rabbit polyclonal GFAP antibody (G4546), and mini protease inhibitor cocktail were purchased from Sigma-Aldrich (USA). Mouse monoclonal Arc antibody (sc-17839) and goat polyclonal BDNF antibody (sc-33904) were purchased from Santa Cruz Biotechnology, USA. Horse radish peroxidase conjugated secondary antibodies were purchased from Bangalore Genei, India, and polyvinyl difluoride (PVDF) membrane was procured from Millipore (Germany). CDRI-08 extract of* B. monniera* was generously provided by Lumen Marketing Company, Chennai. All other biochemicals were purchased from Merck (Germany).

### 2.2. Animals

Young (8 ± 2 weeks) male mice of Swiss albino strain were maintained at 12:12 h light/dark schedule with* ad libitum* standard mice feed and potable water in the animal house of Department of Zoology, Banaras Hindu University, Varanasi. All the animal experiments were approved by the Institutional Animal Ethical Committee, Banaras Hindu University, Varanasi, India. Animals were intraperitoneally injected with normal saline (vehicle) or 3 mg/kg BW scopolamine hydrobromide (dissolved in normal saline) whereas Tween-80 (vehicle) or 120 mg/kg BW CDRI-08 extract (suspended in 5% Tween-80) was administered orally through an oral gavage in the early hours of day (around 9 a.m.) daily for a week.

### 2.3. Treatment Schedule

All the drugs solutions were prepared just prior to use. In the initial experiments, mice were tested for ruling out any possible effect of treatment with vehicles alone and categorized into three groups: untreated (naïve), normal saline injected, and Tween-80 administered group. After obtaining the preliminary results from pilot study, we categorized mice into five groups: (a) SA-mice administered with normal saline, (b) SC-mice injected with scopolamine hydrobromide, (c) SC+CDRI-08 mice injected with scopolamine followed by CDRI-08 after 1 h, (d) CDRI-08-mice treated with CDRI-08 alone, and (e) CDRI-08+SC mice treated with CDRI-08 followed by scopolamine after 1 h. The drug treatment was continued for a week. On the seventh day, mice were sacrificed by cervical dislocation and cerebrum was removed quickly on ice. The biochemical assay of acetyl cholinesterase activity was performed after 3 h of the final drug administration to mice, whereas remaining brain samples were stored at −80°C for further use.

### 2.4. Acetyl Cholinesterase Assay

Acetyl cholinesterase (AChE) assay was done based on the principle and protocol as described earlier by Ellman et al. [27] with some minor modifications. Briefly, the cerebrum from each mouse was weighed and 20% homogenate was prepared in 0.1 M phosphate buffer (pH 8). Then 2.6 mL phosphate buffer (0.1 M, pH 8), 100 *μ*L DTNB, and 0.4 mL homogenate were added in a glass cuvette and mixed thoroughly.* A*_412_ was measured using a UV-VIS spectrophotometer till the absorbance reached a constant value (basal reading). Finally, 20 *μ*L of acetyl thiocholine iodide was added as substrate and change in absorbance was noted at the interval of 2 min to calculate the change in absorbance per minute. The AChE activity was determined by following formula: *R* = 5.74 × 10^−4^ × Δ*A*/*C*, where *R* = rate of enzymatic activity (in moles of acetyl thiocholine hydrolyzed/min/g tissue), Δ*A* = change in absorbance/min, and *C* = concentration of the tissue homogenate (mg/mL).

### 2.5. Semiquantitative Reverse-Transcriptase Polymerase Chain Reaction (RT-PCR)

Total RNA was isolated from the cerebrum of mice using TRI reagent kit. It was estimated by measuring absorbance at 260 nm and purity was checked by *A*_260_/*A*_280_ ratio. Total RNA from different experimental groups was resolved on 1% agarose containing ethidium bromide to check the integrity of RNA by 18S and 28S rRNA. The total RNA was reverse-transcribed to cDNA and PCR-amplified using gene specific primers. The signals were scanned by Alpha Imager system and analyzed by AlphaEaseFC software (Alpha Innotech Corp., USA). The primer pairs and PCR conditions mentioned earlier are summarized in Table 1.

### 2.6. Immunoblotting

The cerebrum was removed from the mice of different experimental groups. The 10% protein lysate for Arc detection was prepared in RIPA buffer, whereas the same was prepared for BDNF and GFAP detection in another homogenizing buffer (50 mM Tris-Cl, pH 7.4, 1 mM EDTA, 120 mM NaCl, and 10 *μ*g complete mini protease inhibitor cocktail). All lysates were centrifuged at 10,000 ×g for 15 min. The supernatant was collected and protein was estimated by Bradford (1976) method. Protein was resolved by 10% SDS-PAGE, followed by semidry electroblotting onto PVDF membrane.

The membrane was blocked in 5% (w/v) fat-free skimmed milk in PBS (pH 7.4) at room temperature for 2 h, incubated overnight at 4°C with mouse anti-Arc polyclonal antibody (1 : 1000), goat anti-BDNF polyclonal antibody (1 : 5000), or rabbit anti-GFAP polyclonal antibody (1 : 1000); washed thrice in 1x PBS, incubated for 2 h at room temperature with HRP-conjugated goat anti-mouse IgG (1 : 1000) for Arc, rabbit anti-goat IgG (1 : 3000) for BDNF, and goat anti-rabbit IgG (1 : 2000) for GFAP; and washed twice in 0.1% PBST and signal was detected with ECL reagents on a X-ray film. For loading control, the same membrane was probed for *β*-actin with HRP-conjugated anti-*β*-actin antibody (1 : 10,000) for 3 h at room temperature.

### 2.7. Statistical Analysis

Each experiment was repeated thrice (*n* = 3 × 3 mice/group). For AChE assay, the rate of enzymatic activity was measured as *μ*moles of acetyl thiocholine hydrolyzed/min./g tissue weight. For RT-PCR, the signal intensity of each candidate message was normalized against the signal intensity of internal control, that is, GAPDH. For immunoblotting, the signal intensity of each candidate protein was analyzed after normalization against the signal intensity of *β*-actin. The data are presented as a histogram with the mean (±SEM) of three values calculated as relative density values. Statistical analysis was performed using one-way analysis of variance (ANOVA) followed by Tukey's post hoc test through SPSS for Windows (standard version 16.0). *P* values ≤0.05 were considered significant.

## 3. Results

### 3.1. The Drug Vehicles* Per Se* Did Not Affect Expression of Plasticity Markers

**Figure 1:**
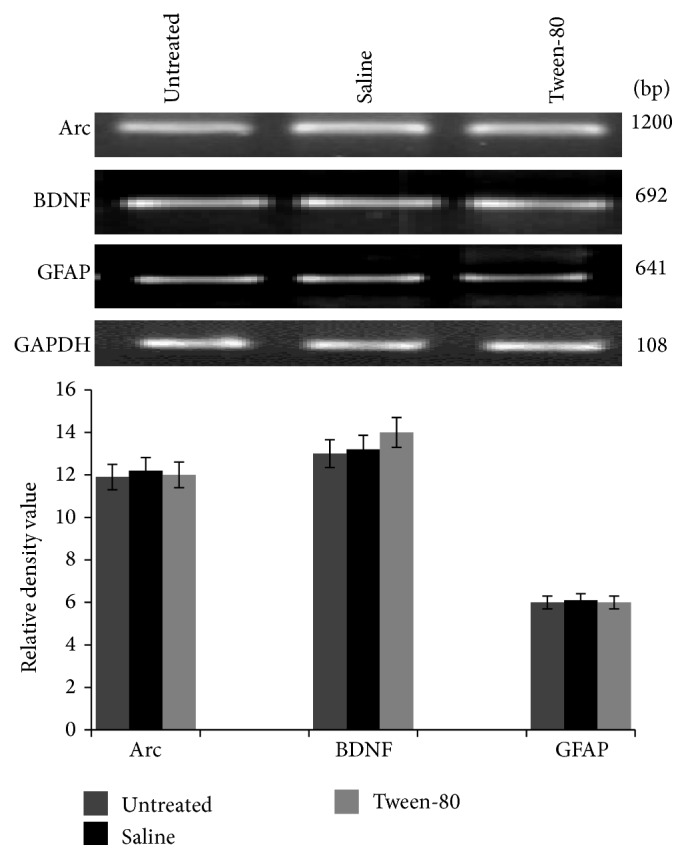
RT-PCR analysis of Arc, BDNF, and GFAP in vehicle control groups of male mice. Signal intensity for Arc, BDNF, and GFAP was normalized against the signal intensity for GAPDH individually. The data are presented as a histogram with the mean (±SEM) of three values calculated as relative density values of Arc/GAPDH, BDNF/GAPDH, and GFAP/GAPDH.

In order to rule out the* per se* effect of vehicle controls for the drugs, we analyzed their effect on gene expression as compared to untreated naïve control. We observed no significant difference in the expression of BDNF, Arc, and GFAP mRNA among naïve (untreated), 0.9% saline (vehicle for scopolamine hydrobromide), and 5% Tween-80 (vehicle for CDRI-08) treated groups (Figure 1). Therefore, only saline control group was used for further experiments.

### 3.2. CDRI-08 Extract Attenuated Scopolamine Mediated Increase in AChE Activity in Mouse Cerebrum

**Figure 2:**
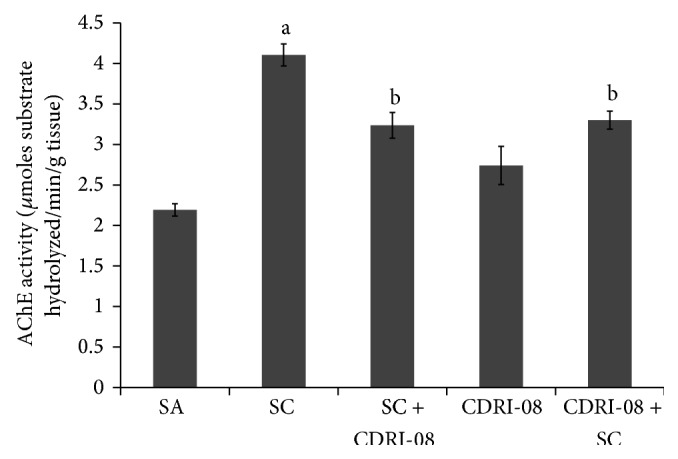
Effect CDRI-08 on AChE activity in the cerebrum of scopolamine (SC) administered amnesic mice. AChE activity plotted as mean AChE activity ± SEM (*μ*moles substrate hydrolysed/min./g tissue wt.) in three cerebrum samples in each experimental group. a denotes *P* ≤ 0.05, significantly different from control (SA) group; b denotes *P* ≤ 0.05, significantly different from scopolamine (SC) group.

The rate of degradation of acetylcholine was determined by AChE assay and we found that scopolamine hydrobromide increased the activity of AChE by 2-fold as compared to control. Pre- and posttreatment with CDRI-08 extract decreased AChE activity as compared to scopolamine (1.25-fold). However, with the* per se* treatment of CDRI-08, we observed insignificant difference in the activity of AChE as compared to saline treated group (Figure 2).

### 3.3. Expression of BDNF, Arc, and GFAP mRNA Was Upregulated upon Administration of CDRI-08 Extract in the Cerebrum of Scopolamine Induced Amnesic Mice

**Figure 3:**
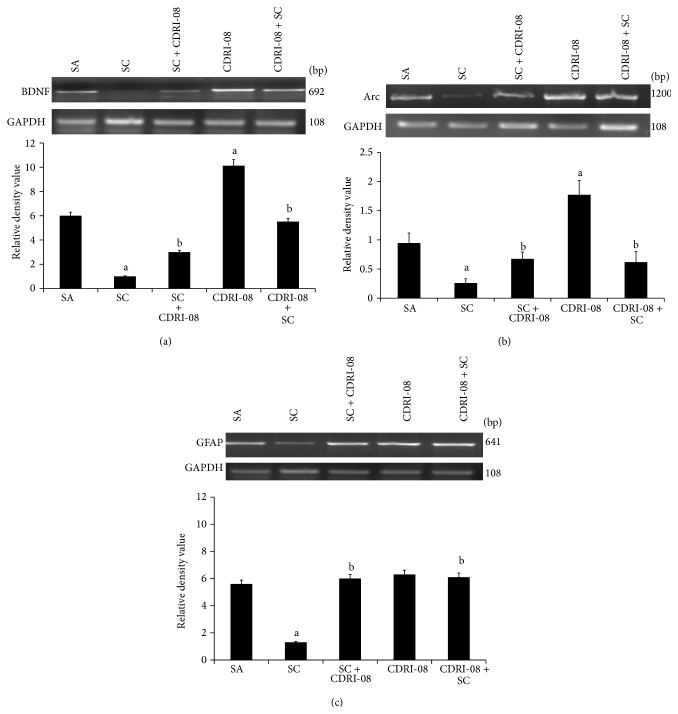
Effect of CDRI-08 on the mRNA expression of (a) BDNF, (b) Arc, and (c) GFAP in the cerebrum of SC administered amnesic mice. The data are presented as a histogram with the mean (±SEM) of three values calculated as relative density values of BDNF/GAPDH, Arc/GAPDH, and GFAP/GAPDH. a denotes *P* ≤ 0.05, significantly different from control (SA) group; b denotes *P* ≤ 0.05, significantly different from SC group.

Scopolamine drastically reduced BDNF mRNA level (6.25-fold) in mouse cerebrum. Pretreatment with CDRI-08 markedly attenuated the decrease by 5-fold while posttreatment attenuated the decrease by 3-fold as compared to scopolamine. The extract alone increased the level of BDNF mRNA by 1.3-fold as compared to saline (Figure 3(a)). Similar to BDNF, Arc mRNA was significantly downregulated by scopolamine (3.33-fold) and pre- as well as posttreatment with CDRI-08 showed 3-fold increase in Arc mRNA expression as compared to scopolamine (Figure 3(b)). The Brahmi extract alone also extensively upregulated (about 2-fold) the expression of Arc mRNA level as compared to control. Similar to neuronal markers, GFAP mRNA was significantly reduced by 4.16-fold by scopolamine treatment. Pre- and posttreatment with CDRI-08 equally attenuated the decrease by 4-fold as compared to scopolamine, whereas treatment with the extract alone did not show significant alteration as compared to saline control (Figure 3(c)).

### 3.4. Effect of CDRI-08 on the Protein Expression of BDNF, Arc, and GFAP Was Similar to That of mRNA

**Figure 4:**
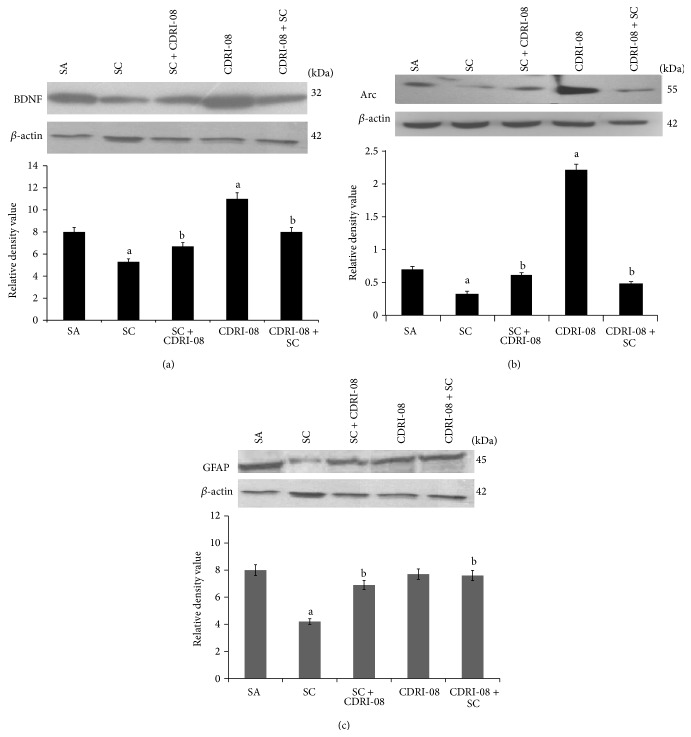
Effect of CDRI-08 on the protein expression of (a) BDNF, (b) Arc, and (c) GFAP in the cerebrum of SC administered amnesic mice. The data are presented as a histogram with the mean (±SEM) of three values calculated as relative density values of BDNF/*β*-actin, Arc/*β*-actin, and GFAP/*β*-actin. a denotes *P* ≤ 0.05, significantly different from control (SA) group; b denotes *P* ≤ 0.05, significantly different from scopolamine (SC) group.

Scopolamine reduced the expression of 32 kDa BDNF protein (1.6-fold). Similar to mRNA, pretreatment with CDRI-08 extract attenuated the decrease of BDNF protein by 1.5-fold and posttreatment attenuated the decrease by 1.3-fold as compared to scopolamine. Treatment with CDRI-08 extract alone also upregulated the expression of BDNF protein (1.3-fold) (Figure 4(a)). Scopolamine administration significantly downregulated (1.75-fold) the expression of 55 kDa Arc protein in the cerebrum as compared to saline injected mice. Both post- and pretreatment with CDRI-08 augmented Arc protein level in a significant manner (2.8-fold) as compared to scopolamine injected group. The administration of extract alone significantly upregulated (3.3-fold) the expression of Arc protein (Figure 4(b)). Corresponding to neuronal markers, expression of 45 kDa GFAP protein was reduced by 2-fold by scopolamine treatment. Pre- and posttreatment with CDRI-08 extract attenuated the decrease by 1.8- and 1.6-fold, respectively. The extract* per se* did not alter GFAP protein expression (Figure 4(c)).

## 4. Discussion

*B. monniera* is recognized as a memory-enhancing agent in the centuries-old Ayurvedic literature and its beneficial effects have been endorsed to the active constituent saponin, as bacosides A, bacoside B, and bacosaponins. However the molecular details are still undefined.

In the present study, we have explored the recovery potential of bacoside-A enriched alcoholic extract CDRI-08 [28] in scopolamine induced amnesic mouse model emphasizing molecular markers of brain plasticity. Our study showed that CDRI-08 extract significantly attenuated scopolamine induced downregulation of neuronal and glial plasticity markers and markedly elevated their expression level, indicating enhancement of brain plasticity.

Scopolamine is an acetylcholine (ACh) receptor antagonist blocking cholinergic neurotransmission and causing memory impairment. The concentration of ACh is regulated by a serine hydrolase known as acetyl cholinesterase (AChE), which hydrolyses and inactivates acetylcholine at the synapse [29]. Therefore, we first analyzed the effect of scopolamine on AChE activity and assessed the influence of CDRI-08 treatment in mouse cerebrum. Scopolamine significantly increased the activity of AChE, implying rapid degradation of available ACh and blockade of downstream signaling. Increased AChE levels were also found in the brain of neurodegenerative transgenic mice with impaired memory [30], supporting our result of elevated AChE levels during scopolamine induced amnesia. We observed that CDRI-08 extract decreased AChE activity when administered orally before or after the scopolamine administration. The anticholinesterase activity of the extract might be attributed to its antiamnesic potential as also reported for other* B. monniera* extracts [31].

However, we did not find any significant effect of the extract* per se* on AChE activity as compared to control. As herbal extracts elicit their action slowly with prolonged effects, it is likely that chronic treatment of Brahmi extract with longer duration might deliver anticipated results.

CDRI-08 extract significantly enhanced the expression of plasticity markers in the cerebrum of scopolamine administered amnesic mice, the effect being more pronounced for neuronal genes BDNF and downstream effector Arc than astrocytic GFAP. CDRI-08 administered alone or in combination with scopolamine upregulated both BDNF mRNA and protein level. It has been established that BDNF is controlled by Ca^2+^ regulated transcription factor CREB that remains bound to BDNF promoter in an inactive form. Phosphorylation of CREB by calcium-regulated kinase cascades stimulates the recruitment of components of the basal transcription machinery to BDNF promoter, and then new BDNF mRNA is synthesized [32, 33]. Earlier reports on* B. monniera* root extract reveal that it influences kinase-CREB pathway to ameliorate memory impairment. Recently, Preethi et al. [34] reported that CDRI-08 extract improves hippocampus-dependent contextual memory with concomitant activation of ERK-CREB signaling cascade.

We also noted a prominent increase in the cerebrum expression of Arc mRNA and protein by CDRI-08 extract during scopolamine induced amnesia. The induction of the IEG, Arc, is strongly implicated in synaptic plasticity [35, 36]. Overexpression of Arc resulted in enhanced AMPA receptor endocytosis and increased LTP and memory consolidation [37]. However, the regulatory mechanisms underlying the activity-dependent transcription of Arc and related neuronal functions remain largely unknown. Recent investigations demonstrate that BDNF-dependent Arc transcription is crucial for neuronal plasticity [38–40]. Therefore, upregulation of BDNF by CDRI-08 might activate Arc transcription and translation during scopolamine induced amnesia.

Interestingly, CDRI-08 extract also attenuated scopolamine mediated decrease in astrocytic plasticity marker, GFAP. However, GFAP expression was unaltered upon* per se* treatment with the extract. GFAP regulates metabolism and activity of the neurons and maintains neuron-glia interactions and plastic rearrangement of the synaptic connections [41]. The excessive increase in GFAP expression resulting in astrogliosis implicates inflammation, increase in reactive oxygen species, and transition to neurodegenerative state [42]. Therefore, our findings suggest the amazing neuroprotective property of CDRI-08 extract which is balanced enough to maintain glial plasticity providing neuronal support but not exceeding the threshold such that it becomes neurotoxic. GFAP gene regulation is also relatively less studied and few reports [43] propose the involvement of transcription factors pCREB and NF*κ*B in regulation of transcription of GFAP. Overall, our data suggest that CDRI-08 extract might activate cholinergic signaling, downstream kinase cascades, and eventually CREB mediated basal transcriptional machinery of memory linked neuronal and glial plasticity markers.

## 5. Conclusion

Our findings provide the first evidence on molecular basis of recovery potential of CDRI-08 extract in scopolamine induced amnesia in relation to neuronal and glial plasticity markers. Further investigations on detailed pathways and morphological analysis of neurons and glia are warranted to establish the extract as a potential therapeutic target in memory disorders.

## Figures and Tables

**Table 1:** Primer sequences and PCR conditions.

Gene	Sequence of forward and reverse primers (5′→3′)	Condition of (a) denaturation (b) annealing (c) extension	Number of cycles
Arc [17]	FP-GGCGACCAGATGGAGCTGGACCATA- RP-CTGGCCCCTCTATTCAGGCTGGGTC-	(a) 94°C, 1 min (b) 59°C, 1 min (c) 72°C, 1.5 min	35

BDNF [20]	FP-TGCCAGAGCCCCAGGTGTGA- RP-CTGCCCTGGGCCCATTCACG-	(a) 94°C, 1 min (b) 63°C, 30 sec (c) 72°C, 45 sec	32

GFAP [20]	FP-TTCCTGTACAGACTTCTCC- RP-CCCTTCAGGACTGCCTTAGT-	(a) 94°C, 1 min (b) 52°C, 30 sec (c) 72°C, 45 sec	29

GAPDH [20]	FP-GTCTCCTGCGACTTCAG- RP-TCATTGTCATACCAGGAAATGAGC-	(a) 94°C, 1 min (b) 52°C, 30 sec (c) 72°C, 30 sec	26
